# What has reproductive health decision-making capacity got to do with unintended pregnancy? Evidence from the 2014 Ghana Demographic and Health Survey

**DOI:** 10.1371/journal.pone.0223389

**Published:** 2019-10-10

**Authors:** Bright Opoku Ahinkorah, Abdul-Aziz Seidu, Francis Appiah, Linus Baatiema, Francis Sambah, Eugene Budu, Edward Kwabena Ameyaw

**Affiliations:** 1 The Australian Centre for Public and Population Health Research, Faculty of Health, University of Technology Sydney, NSW, Sydney, Australia; 2 Department of Population and Health, College of Humanities and Legal Studies, University of Cape Coast, Cape Coast, Ghana; 3 Department of Health, Physical Education, and Recreation, University of Cape Coast, Cape Coast, Ghana; Anglia Ruskin University, UNITED KINGDOM

## Abstract

**Introduction:**

Women’s reproductive health decision-making is indispensable for improving their reproductive health and achieving Sustainable Development Goal three. This study explored the association between reproductive health decision-making capacity and unintended pregnancy among women in Ghana.

**Materials and methods:**

We used data from the 2014 version of the Ghana Demographic and Health Survey. The unit of analysis for this study was pregnant women at the time of the survey (679). Bivariate and multivariable analyses were conducted using Pearson chi-square tests and binary logistic regression respectively.

**Results:**

We found that women who had the capacity to make reproductive health decision [AOR = 0.61; CI = 0.51–0.89] were less likely to experience unintended pregnancies, compared to those who did not have the capacity. Age was found to have a statistically significant influence on unintended pregnancy, with women aged 25–29 years [AOR = 0.29; CI = 0.13–0.63], 30–34 years [AOR = 0.18; CI = 0.08–0.45], and 35–39 years [AOR = 0.26; CI = 0.10–0.68] being less likely to experience unintended pregnancy compared to those aged 15–19 years. Women with primary level of education were more likely to have unintended pregnancies, compared to those with no education [AOR = 2.07; CI = 1.12–3.84].

**Conclusion:**

This study has filled the gap in the already existing literature on the association between reproductive health decision making capacity and unintended pregnancy in Ghana and has created a room for specific interventions geared towards reducing unintended pregnancies, especially among women who are not capable of making reproductive health decisions, women aged 15–19 years, those with primary education, Traditionalists and unmarried women.

## Introduction

Unintended pregnancies connote pregnancies that are not wanted or those that are mistimed at the time of conception [1, 2]. Although almost 41 out of every 100 pregnancies were considered unintended between 2010 and 2014 worldwide, the global statistics on unintended pregnancy shows substantial regional variations [3]. While unintended pregnancy rate declined by 30 percent in high-income countries among women aged 15–44 years between 1990–94 and 2010–14, low- and middle-income countries only made a petite decline. Sub-Saharan Africa only recorded 16 percent reduction [3]. On the part of Ghana, reported prevalence of unintended pregnancy is between 29.8 to 70 percent [2; 4]. While Eliason et al. [2] noted 70 percent of unintended pregnancies among women in Ghana, the 2014 Ghana Demographic and Health Survey also recorded 29.8 percent [4]. Therefore, it is obvious that the phenomenon is still significant in Ghana.

Documented evidence suggest a number of negative complications associated with unintended pregnancies [5–7]. Unintended pregnancies have adverse effects on women’s quality of life and also threatens households’ economic status [5,6]. Additionally, induced abortion and associated problems are mutual consequences of unintended pregnancies [8]. Most of the unintended pregnancies occur in low and middle income countries largely due to high illiteracy, and lack of knowledge and access to contraceptive methods [9]. As a result, allowing women to decide, access and enjoy their full reproductive health and right would be beneficial to offset unintended pregnancies as envisioned by the 174-member states during the International Conference on Population and Development (ICPD) and affirmed by the Fourth World Conference on Women in 1995 [10, 11].

Decision-making as a process commences with identifying a problem, evaluating and selecting an appropriate solution from the alternative solutions [12]. In relation to women reproductive health, decision-making has been explained in the context of decision-making on sexual intercourse and decision-making on condom use [13, 14]. Such decisions are essential because sexual interactions often encompass complex, iterative sexual decision-making processes that challenge women to make a multitude of situational appraisals and engage in a variety of sexual behaviors [15]. According to Prata, Tavrow and Upadhyay [16] and McReynolds-Pérez [17], women’s decision making is essential in their reproductive choices because the choices they make on their reproductive health are critical for them to live a better life. Studies have shown that in countries where women have issues with reproductive health decision-making, unwanted pregnancies and unsafe abortions are high [18, 19]. In this regard, Uberoi and De Bruyn [20] argued that reproductive rights are central to women’s self-determination. Grover [21] also asserted that it is important to have laws that require that women are free to make personal decisions without interference from the state, especially in an important and intimate area such as sexual and reproductive health.

Although the right of women, of which reproductive health forms part, as affirmed by ICPD in 1994 and the Fourth World Conference on Women in 1995 [10,11] has been challenged owing to diverse socio-cultural contexts in sub-Saharan Africa [22]. Women’s ability to make choices about matters pertaining to their reproductive health is often restricted by economic, social and cultural conditions prevailing in the region [13]. In Ghana, Darteh, Dickson and Doku [14] observed that women from rural areas, those without formal education, Muslims, non-working women and women whose partners had no formal education were less likely to make decisions about their reproductive health. This could possibly incline such women towards greater chances of unintended pregnancies and associated health risks. Again, traditional African inheritance system (especially patriarchy) coupled with gender inequalities about contraceptive use also complicates women's reproductive health decision-making capacity [23,24]. In most cases, women have to seek their partners’ approval about matters affecting their reproductive rights and decisions, such as the timing of conception [23,24]. For instance, Ameyaw et al. [25] realised that women who were not deciding independently on their own healthcare, large purchases and visiting family members were less probable to use contraceptives in Ghana. This implies that, even if women are aware of the benefits of regulating their pregnancy, it might be difficult to enforce it.

Several scholars in Ghana have identified numerous predictors of unintended pregnancy. For instance, Nyarko [26] identified age, level of education, marital status, parity and region of residence as significant predictors of unintended pregnancies. Eliason et al. [2] indicated that women not living with their partners were more likely to have unintended pregnancies. Ameyaw [25] also identified age, wealth status, educational level, religion, parity and occupation as influencing unintended pregnancies among women. A similar study conducted by Grindlay et al. [27] identified that women were more likely to experience an unintended pregnancy if they had ever given birth, their sexual debut was earlier or they had 3–4 lifetime sexual partners. Despite the high rates of unintended pregnancies in Ghana [25,26], and the scholarly affirmation that women reproductive health decision making influences their reproductive health outcome, none of the studies that have been conducted on determinants of unintended pregnancies in Ghana have explored the link between reproductive health decision making capacity and unintended pregnancies. Therefore, this study is motivated to bridge this gap by exploring the association between reproductive health decision-making capacity and unintended pregnancy among women in Ghana.

The paper is divided into five sections. The first section presents the introduction of the study. In the materials and methods, we introduce the real data source, methodology, definition of variables–outcome and independent, validity issues, statistical analysis as well as ethical approval for the study. In the results section, we presented both the descriptive and inferential results obtained using the statistical approaches employed to analyse the data. In the discussion, the results are discussed and synthesized with previous evidence, limitations and strength of the study are also acknowledged. The conclusion presents summary remarks and policy implications of the findings obtained.

## Materials and methods

### Real data source

This study used data from the 2014 Ghana Demographic and Health Survey (GDHS). GDHS is a nationally representative population-based survey. The 2014 GDHS is the current and the sixth in a series of Demographic and Health Surveys conducted in Ghana in 1988, 1993, 1998, 2003, and 2008. Similar to the previous surveys, the 2014 GDHS sought to provide current information on fertility and childhood mortality levels; fertility preferences; awareness, approval, and use of family planning methods; maternal and child health; knowledge and attitudes toward HIV/AIDS and other sexually transmitted infections (STI); and prevalence of HIV among the adult population for the country as a whole, for urban and rural areas separately, and for each of the then 10 geographical regions in Ghana.

### Methodology

The 2014 GDHS followed a two-stage sample design and was intended to allow estimates of key indicators at the national level as well as for urban and rural areas and each of the then 10 administrative regions. The first stage was characterized by the selection of 427 clusters consisting of enumeration areas (EAs) delineated for the 2010 Population and Housing Census (PHC). These clusters were selected from the erstwhile10 administrative regions of the country and across urban (n = 216) and rural (n = 211) areas. The 427 EAs were selected with probability proportional to the EA size and with independent selection in each sampling stratum. The EA size is the number of residential households residing in the EA enumerated in the 2010 PHC. A household listing operation was carried out in all the selected EAs, and the resulting lists of households served as a sampling frame for the selection of households in the second stage. To minimize the task of household listing for EAs with more than 200 households, each large EA was segmented. Only one segment was selected for the survey with probability proportional to the segment size. Household listing was conducted only in the selected segment. Therefore, a 2014 GDHS cluster is either an EA or a segment of an EA. The second stage involved the selection of about 30 households from each cluster, constituting a total sample size of 12,831 households. The survey interviewers visited and interviewed only the selected households. No replacements or changes of the selected households were allowed during data collection, in order to prevent bias. All women age 15–49 who were usual members of the selected households or who spent the night before the survey in the selected households were eligible for the female survey. In half of the selected households, all men age 15–49 who were usual members of the households or who spent the night before the survey in the households were eligible for the male survey. The procedures followed in the collection of the data was validated and characterized by various quality control measures.

Data collection was carried out using three pretested and validated questionnaires: household questionnaire, woman's questionnaire, and man's questionnaire. The household questionnaire was used to list all the members and visitors to the selected households, with the information gathered used to identify women and men eligible for individual interviews. Eligible participants had to be permanent residents of the selected households or visitors who stayed in the household the night before the survey. In half of the selected households, the man’s questionnaire was administered to all men age 15–59. The information collected was similar to that of the woman’s questionnaire but was shorter because it did not contain a detailed reproductive history or questions on maternal and child health. The woman’s questionnaire was used to collect information from all eligible women aged 15–49 years. A total of 9,396 women (response rate, 97.3%) were interviewed for the survey. The survey provides a complete birth history of women. As a result, it serves as the best source of data for this study. The unit of analysis for this study is individual pregnant women at the time of the survey (679). As a result, only data concerning them were extracted for this study. A short proposal was written to MEASURE DHS explaining the motive of the study and requesting access to use the dataset. Access to the dataset was granted and was freely downloaded from: https://dhsprogram.com/what-we-do/survey/survey-display-437.cfm.

### Definition of study variables

#### Discussion on outcome variable

The outcome variable was unintended pregnancy. During the survey, respondents were asked: "When you got pregnant, did you want to get pregnant at that time?” The responses to this question were either “Yes” = 1 or “No” = 0.

#### Discussion on explanatory variables

The main explanatory variable was reproductive health decision-making capacity. It was derived from two variables: decision-making on sexual intercourse and decision-making on condom use. For decision-making on sexual intercourse, women were asked whether they can refuse their partners sex while for decision-making on condom use, women were asked whether they can ask their partners to use condoms during sexual intercourse. Reproductive health decision-making capacity was generated from the combination of decision-making on sexual intercourse and decision-making on condom use variables. Following previous studies on reproductive health decision-making [see 13, 14], the original response category of these variables (1 = yes, 2 = no and 3 = don’t know/ not sure) were re-categorised, whereby “no and don’t know/not sure” were recoded as “0”, with “yes” recoded as “1”. Responses coded as “0” were considered as not capable in making reproductive health decision whilst those coded as “1" were labeled as capable of making reproductive health decisions.

The other explanatory variables were the age of respondents, wealth status, highest educational level, religion, marital status, ethnicity, residential status, region of residence, parity, work status, and intention to use contraception. These independent variables have been included in this study primarily based on conclusions drawn on them from previous studies [2,26,28–29] to be significantly associated with unintended pregnancy. Age was categorized into 7 age groups: 15–19, 20–24, 25–29, 20–34, 35–39, 40–44, and 45–49. Wealth status was categorised as poor, middle and rich. Educational level was classified into four categories: no education, primary education, secondary education, and higher education. Religion was categorised as Christianity, Islam, Traditionalist, and No religion. Marital status was recoded as not married and married. Ethnicity was coded as Akan, Ga/Adangbe, Ewe, Mole-Dagbani and Other. Type of residence was coded as urban or rural. Region was captured as Western, Central, Greater Accra, Volta, Eastern, Ashanti, Brong-Ahafo, Northern, Upper East, and Upper West. Parity was recoded as 0, 1–3, and 4 and above, taking into consideration Ghana’s current total fertility rate of 4.2 [4]. Working status was categorised as working and not working, while the intention to use contraception was captured as intends to use later, and does not intend to use.

### Validity issues

The 2014 GDHS was able to minimize selection bias due to the sampling strategy which resulted in a high response rate (97.3%) for women. In addition, the survey employed standardized pretested data collection instruments and procedures. There was also extensive training of interviewers that guaranteed the collection of reliable information. Missing data was not a concern in this study. Of the variables of interest, none had missing responses.

### Statistical analysis

Descriptive and inferential statistics were conducted. Descriptive figures were presented in percentages. Bivariate analyses were conducted using Pearson chi-square tests (see Table 1). Using the explanatory variables which were significantly associated with unintended pregnancy among women aged 15–49 from the chi-square test, two binary logistic regression models were developed to establish the specific attributes of the significant explanatory variables which contributed to unintended pregnancy among the women (see Table 2). The logistic regression model assumed that the dependent variable should be dichotomous and the individual should have a probability of 0.5 of choosing either of two alternatives. Here the study is interested in assessing the probability of having unintended pregnancy. The outcome variable (unintended pregnancy) has two categories. This is represented by a binary random variable *Y_i_* that takes the value of one if an individual is having an unintended pregnancy and zero otherwise, as follows: $Y_{i}=\left\{ \begin{array}{c} 1=Yes\\ 0=No \end{array}\right.$

**Table 1:** Prevalence of unintended pregnancy among Ghanaian women by reproductive health decision-making and socio-demographic characteristics (*N* = 679).

Variable	Sample		Intended Pregnancy	Unintended Pregnancy	X^2^(P-Value)
**Reproductive health decision-making capacity**	**N**	**%**	%	%	
Incapable	204	30.0	55.6	44.4	**9.5(p<0.01)
Capable	475	70.0	67.9	32.1	
**Age**					***31.1(p<0.001)
15–19	64	9.4	35.0	65.0	
20–24	123	18.1	62.5	37.5	
25–29	178	26.3	70.6	29.4	
30–34	174	25.7	71.1	29.9	
35–39	103	15.2	65.6	34.4	
40–44	30	4.4	52.9	47.1	
45–49	7	1.0	57.9	42.9	
**Wealth status**					**14.1(p<0.01)
Poor	249	36.7	62.5	37.5	
Middle	140	20.6	54.0	46.0	
Rich	290	42.8	72.9	27.1	
**Highest education level**					***31.1(p<0.001)
No education	161	23.8	77.1	22.9	
Primary/JHS	113	16.6	49.6	50.4	
Secondary/SHS	358	52.7	56.7	40.3	
Higher/tertiary	47	6.9	76.7	23.3	
**Religion**					***23.5(p<0.001)
Christianity	542	79.9	59.8	40.2	
Islam	103	15.2	81.7	18.3	
Traditionalist	19	2.8	54.5	45.5	
No Religion	15	2.3	66.7	33.3	
**Marital status**					***60.7(p<0.001)
Not married	277	40.8	45.1	54.9	
Married	402	59.2	74.9	25.1	
**Ethnicity**					***54.0(p<0.001)
Akan	330	48.6	57.7	42.3	
Ga-Dangme	30	4.4	58.3	41.7	
Ewe	109	16.1	39.8	60.2	
Mole-Dagbani	99	2.4	79.0	21.0	
Others	110	28.5	75.6	24.4	
**Residential status**					**7.4(P<0.01)
Urban	337	49.7	69.7	30.3	
Rural	341	50.3	59.7	40.3	
**Region**					***79.3(p<0.001)
Western	73	10.7	71.0	29.0	
Central	75	11.1	53.4	46.6	
Greater Accra	134	19.7	62.5	37.5	
Volta	45	6.6	28.3	71.7	
Eastern	71	10.4	46.7	53.3	
Ashanti	107	15.7	56.4	43.6	
Brong Ahafo	60	8.8	60.0	40.0	
Northern	71	10.5	86.0	14.0	
Upper East	29	4.3	82.1	17.9	
Upper West	15	2.2	81.2	18.8	
**Parity**					***18.0(p<0.001)
0	144	21.2	57.0	43.0	
1–3	380	55.9	71.2	28.8	
4 and above	155	22.9	54.4	45.6	
**Working status**					**6.1(p<0.01)
Not working	135	19.9	55.1	44.9	
Working	544	80.1	66.5	33.5	
**Contraceptive use intention**					**8.0(p<0.01)
Intends to use later	397	58.5	60.0	40.0	
Does not intend to use	281	41.5	70.7	29.3	

* *p* < 0.05

** *p* < 0.01

*** *p* < 0.001

**Table 2:** Logistic regression on correlates of unintended pregnancy among women in Ghana.

Variable	Model I OR (95%CI)	Model II AOR (95%CI)
**Reproductive health decision-making capacity**		
Incapable	Ref	Ref
Capable	0.59**[0.42–0.83]	0.61**[0.51–0.89]
**Age**		
15–19	Ref	Ref
20–24	0.32***[0.17–0.61]	0.47*[0.23–0.97]
25–29	0.22***[0.12–0.42]	0.29**[0.13–0.63]
30–34	0.22***[0.17–0.41]	0.18***[0.08–0.45]
35–39	0.28***[0.14–0.55]	0.26**[0.10–0.68]
40–44	0.48[0.20–1.13]	0.35[0.10–1.18]
45–49	0.40[0.08–1.98]	0.26[0.04–1.54]
**Wealth status**		
Poor	1.42[0.95–2.13]	1.28[0.74–2.23]
Middle	0.62[0.43–0.90]	0.73[0.36–1.51]
Rich	Ref	Ref
**Highest education level**		
No education	Ref	Ref
Primary	3.43***[2.10–5.61]	2.07*[1.12–3.84]
Secondary	2.28***[1.53–3.39]	1.74[0.98–3.10]
Higher	1.02[0.47–2.23]	2.49[0.93–6.72]
**Religion**		
Christianity	Ref	Ref
Islam	0.33***[0.21–0.53]	0.63[0.34–1.16]
Traditionalist	1.24[0.53–2.93]	3.51*[1.11–11.05]
No Religion	0.74[0.25–2.21]	0.59[0.16–2.25]
**Marital status**		
Not married	Ref	Ref
Married	0.27***[0.20–0.38]	0.46**[0.28–0.74]
**Ethnicity**		
Akan	Ref	Ref
Ga-Dangme	0.97[0.42–2.27]	0.60[0.20–1.82]
Ewe	2.06**[1.25–3.41]	1.26[0.63–2.52]
Mole-Dagbani	0.36***[0.23–0.56]	1.32[0.60–2.92]
Others	0.44**[0.28–0.70]	1.029[0.50–2.13]
**Residence**		
Rural	Ref	Ref
Urban	0.64**[0.47–0.89]	1.18[0.72–1.95]
**Region**		
Western	Ref	Ref
Central	2.14**[1.07–4.28]	2.74*[1.17–6.40]
Greater Accra	1.47[0.71–3.04]	3.62**[1.40–9.33]
Volta	6.22***[2.72–14.22]	8.40***[2.71–26.09]
Eastern	2.8**[1.40–5.59]	3.43**[1.49–7.89]
Ashanti	1.89[0.92–3.90]	3.42**[1.41–8.32]
Brong Ahafo	1.63[0.81–3.28]	2.25[0.91–5.56]
Northern	0.40*[0.18–0.86]	0.58[0.19–1.76]
Upper East	0.53[0.24–1.21]	0.67[0.22–2.05]
Upper West	0.57[0.23–1.38]	0.78[0.24–2.58]
**Parity**		
0	Ref	Ref
1–3	0.54**[0.36–0.81]	1.22[0.68–2.19]
4 and above	1.11[0.70–1.75]	4.68***[2.07–10.52]
**Working status**		
Not working	Ref	Ref
Working	0.62*[0.42–0.91]	0.70[0.43–1.14]
**Contraceptive use intention**		
Intends to use later	Ref	Ref
Does not intend to use	0.62**[0.45–0.87]	0.57**[0.38–0.85]

* *p* < 0.05

** *p* < 0.01

*** *p* < 0.001 Ref = reference CI = Confidence Interval OR = Odds Ratio

The outcomes of this binary variable occur with probability п_*i*_ which is a conditional probability on the explanatory variables. For an individual identified as having unintended pregnancy as: ${п}_{i}\equiv Pr(Y_{i)\equiv Pr(Y_{i}|X_{i}}$ and thus, the conditional mean equals the probability as follows:

μ*Yi*|*X*_*i* =_ п_*i*_×1+(−п_*i*_)×0 = п_*i*_. For a binary model the conditional distribution of the dependent variable, or random component in a GLM, is given by a Bernoulli distribution. Thus, the probability function of $Y_{i\;is\;PY\;(yi)={\pi i}^{yi}\;(1-}{п}_{i})1-y_{i}$

In the binary model, the conditional mean *μi* is the conditional probability *πi*. The logit of π is the natural logarithm of the odds that the binary variable *Y* takes a value of one rather than zero. In that regard, this gives the relative chances of an individual having an unintended pregnancy or not. The logit model in a linear, additive form for the logarithm of odds is specified as:

$$ln\frac{{п}_{i}}{\left[1-{п}_{i}\right]}=\eta_{i}=\beta_{0}+\beta_{1}X_{ij}+{\ldots \ldots \ldots .+\beta }_{k}X_{ik}$$

For the logistic, the multiplicative model for the odds is specified as:

$$\frac{{п}_{i}}{\left[1-{п}_{i}\right]}=e^{\eta_{i}\prime }=e^{\beta_{0}}(e\beta_{k})xi1+1\ldots \ldots ..(e\beta_{k})xik$$

The conditional probability π_*i*_ is then

$$\pi_{i}=\frac{1}{\left[1+e^{{-\eta }_{i}}\right]}=\frac{1}{\left[1+e^{-}\sum_{j=0}^{k}\beta_{j}X_{ij}\right]}$$

The partial regression coefficients (β_*j*_) are interpreted as marginal changes of either the logit or odds ratios. For logit, thus, the coefficient β_*j*_ indicates the change in the logit due to a one-unit increase in *X_j_*. The empirical logit model for the study is specified as:

$$\begin{array}{l} Unintended\;Prenancy\\ \;=\beta_{0}+\beta_{1}{RHDec\;Cap}_{i}+\beta_{2}{Age}_{i}+\beta_{3}{Wealth\_Stat}_{i}+\beta_{4}{Educ}_{i}+\beta_{5}{Reli}_{i}\\ \;+\beta_{6}{Marit\_Stat}_{i}+\beta_{7}{Ethnic}_{i}+\beta_{8}{Resi}_{i}+\beta_{9}{Pari}_{i}+\beta_{10}{Working}_{i}\\ \;+\beta_{11}{Intent\_Contrace}_{i}+\varepsilon_{i} \end{array}$$

All analysis was done in STATA/SE 14.2 for mac OS (StataCorp LLC, College Station, Texas USA). The choice of the reference categories for the variables were informed by a priori and previous studies [25, 26]. Tests for multicollinearity were also conducted and the results showed that there was no collinearity between the covariates (Mean VIF = 1.62). The fit of our final model was checked using the “svylogitgof” command. The model fit results showed that there was no evidence of a lack of fit of our model in significantly predicting unintended pregnancy. For each variable included in the regression models, odds ratios (ORs) and 95% confidence intervals (95% CI) were calculated.

### Ethical approval

This study was approved by the institutional review board of the Ghana Health Service and the ethics committee of the DHS Program. Informed consent was obtained from all the respondents before the commencement of interviews with each respondent. Data is available on https://dhsprogram.com/what-we-do/survey/survey-display-437.cfm.

## Results

### Chi-square analysis results

Table 1 shows results of the chi-square analysis of the prevalence of unintended pregnancy among Ghanaian women by reproductive health decision-making capacity and socio-demographic characteristics. All the independent variables had statistically significant association with unintended pregnancy through the chi-square tests. The highest prevalence of unintended pregnancy was reported by women living in the Volta Region (71.7%), women aged 15–19 years (65.0%), those of the Ewe ethnic group (60.2%). unmarried women (54.9%), those who resided in the Eastern Region (53.3%), those with secondary/SHS level of education (50.4%), those in the middle wealth status (46.0%), those with parity 4+ (45.6%), those who were Traditionalists (45.5%), those who were working (44.9%), those who were incapable of making reproductive health decisions (44.4%), rural residents (40.3%) and those who had the intention of using contraceptives later (40.0%).

### Logistic regression analysis results

The binary logistic regression analysis conducted among reproductive health decision-making capacity, unintended pregnancy and the other explanatory variables (socio-demographic characteristics) showed significant associations between unintended pregnancy and some of the explanatory variables (reproductive health decision-making capacity, contraceptive use intention, parity, region, marital status, religion, and age) (see Table 2). As shown in model II, women who had the capacity to make reproductive health decisions [AOR = 0.61; CI = 0.51–0.89], women aged 30–34 years [AOR = 0.18; CI = 0.08–0.45] and those who had no intention to use contraceptives [AOR = 0.57; CI = 0.38–0.85] and married women [AOR = 0.46; CI = 0.28–0.74] were less likely to experience unintended pregnancies. On the other hand, women with primary level of education [AOR = 2.07; CI = 1.12–3.84], women who were Traditionalists [AOR = 3.51; CI = 1.11–11.05], women from the Volta Region [AOR = 8.40; CI = 2.71–26.09] and women with 4+ parity [AOR = 4.8; CI = 2.07–10.52] were more likely to experience unintended pregnancies [AOR = 0.57; CI = 0.38–0.85].

## Discussion

In this present study, we examined the association between reproductive health decision-making capacity and socio-demographic factors, on the one hand, and unintended pregnancy among women in Ghana, on the other hand. Our findings on the influence of reproductive health decision-making capacity on unintended pregnancy showed that women who were capable of making reproductive health decisions were less likely to experience unintended pregnancy, compared to those who were not capable of making reproductive health decisions. The negative association between women’s reproductive health decision capacity and unintended pregnancy is consistent with a study by Abada and Tenkorang [28], who argued that regardless of women’s status, having a final say in household and sexual matters with husbands lowers the risk of unwanted births. The finding still corroborates a previous study done in Bangladesh, which indicated that women with low decision-making autonomy are more likely to experience unintended pregnancy [30]. The possible explanation to this is the fact that women who have decision-making capacity are able to fight for their sexual rights [31], in the form of regulating sexual activity and advocating the use of contraceptives when necessary, and this will consequently reduce the tendency of unintended pregnancy. The proportion of unintended pregnancy among women who had decision making capacity was also quite high in this study. There is the possibility that other factors such as contraceptive failure might be accounting for this.

Our study also found a negative relationship between age and unintended pregnancy, indicating that the lower the age of a woman, the higher the chances of unintended pregnancy. Studies by Ameyaw [25] and Nyarko [26] also found a negative relationship between age and unintended pregnancy. The finding that young women are at higher risk of unintended pregnancies has been explained in previous studies by Vázquez-Nava et al. [32], Christofides et al. [33] and Grindlay et al. [27]. They explained that inadequate access to and use of contraceptives, risky sexual behaviour (increasing number of sexual partners and younger age at sexual debut) are the possible pathways through which young women risk unintended pregnancies. The fact that younger women are more likely to experience unintended pregnancy could also be attributed to sexual violence which has been found to be high among young women [34,35]. In addition, factors such as inadequate access to information on sex, lack of knowledge of contraceptive, inability to negotiate safe sex, cultural taboos on discussing sex, fear of stigma and difficulty in access family planning services may explain why unintended pregnancies are high among young women.

Another key finding was how educational level influences women’s experience of unintended pregnancy in Ghana. Our study found that women with no formal education were less likely to have unintended pregnancy, compared to those with some level of formal education. A number of studies have shown similar associations between educational level and unintended pregnancy [36–38]. Similar findings were found also by Ameyaw [25], who identified that women with higher educational qualifications were noted to be more likely to have unintended pregnancy than uneducated women. Although other studies have found that uneducated women are more likely to have unintended pregnancies [36,39], a possible explanation to our finding could be that women with some level of education may be engaged in paid jobs and may consider pregnancy in the course of their employment as unintended, especially when it has negative impact on their productivity.

Further results also showed a relationship between religion and unintended pregnancy. Specifically, women who were Traditionalists were more likely to have unintended pregnancies, compared to Christians. The finding contradicts the findings of Ameyaw [25], who found that women who were Traditionalists were less likely to experience unintended pregnancies as compared to Christian women. Nyarko [26], however, did not find any significant association on the influence of religion on unintended pregnancy in Ghana. Even though the current study shares some resemblance with the studies of Ameyaw [25] and Nyarko [26] in terms of the study area, population and variables, the difference in findings could be explained by the addition of reproductive health decision-making capacity as part of the variables in the current study. The plausible explanation for the relationship between religion and unintended pregnancy could be that most Christians have very strict doctrines, beliefs, and laws regarding early sexual initiation and unwanted pregnancies. Religion has been found to have a strong impact on an individual’s normative orientation towards sex [40] and this has been described by sociologists of religion as constraints on sexual expression, producing greater control over sexual impulses [41,42]. Hitchens [43] also explains that that religion’s relationship to sexuality is basically ‘antagonistic’, and that Christianity, in particular, is against sexual intercourse, more especially when it happens outside marriage.

The study revealed that unmarried women were more likely to experience unintended pregnancies, compared to married women. This finding is consistent with the findings of Eliason, Baiden, Yankey and Awusabo–Asare [2], Exavery et al. [44] and Palamuleni and Adebowale [45] but contradicts the findings of Nyarko [26], who found that married women are more likely to experience unintended pregnancies, compared to unmarried women. A possible explanation for the finding of the current study is that unmarried women are more likely to engage in sexual activity for reasons other than childbearing [44]. Moreover, the absence of inter-partner communication regarding pregnancy as evident among unmarried women increases the risk of unintended pregnancies [39]. Similarly, in a study on the risk factors for unplanned pregnancy among young women in Tanzania, Calvert et al. [36] adduced that one of the possible reasons for the high prevalence of unintended pregnancies among unmarried women could be linked to their engagement in frequent unprotected sex with their casual partners. Such women are similar to women of our study who reported high prevalence of unintended pregnancies because they intended to use contraceptives later. Women who had no intention to use contraceptives may be ready to give birth, hence experiencing a lesser likelihood of unintended pregnancies.

The odds of experiencing unintended pregnancies were high among women in the Central, Greater Accra, Volta, Eastern, and Ashanti regions. These regions are located in the southern sector of Ghana and are beneficiaries of major socio-economic development of the country. Hence, these women might be engaged in employment that requires so much time to the extent that getting pregnant may bring a burden on them and, as a result, they may consider their pregnancies as unintended, especially when they have already had their desired number of children. On the contrary, using a similar population and variable, Nyarko [26] found that unintended pregnancies were low among women in the southern sector of Ghana, and explained that the regions considered to be located in the southern sector of Ghana are most likely to have access to pregnancy prevention information and services, including access to family planning facilities and services, compared to their counterparts in other regions [26]. It is important to note that the role of region of residence in the risk of unintended pregnancy is not well-documented in the literature. However, Palamuleni and Adebowale [45] identified regional differences in unwanted pregnancies in their studies and attributed their findings to variations in the social and economic characteristics of the regions.

Our study further indicated that women with high parity (4 and above) were more likely to experience unintended pregnancies, compared to women who have no child and those with 1–3 children. This finding supports the findings of Fite, Mohammedamin and Abebe [46], who found that pregnant women with parity 3 and above were 20 times more likely to experience unintended pregnancy than pregnant women with parity 1. Habib et al. [37] also found that women who had parity greater than two were more likely to have an unintended pregnancy. This finding is comparable to studies conducted in other low- and middle-income countries [47–49]. The reason for the finding could be that high parity women might already have adequate children and will have less intention of engaging in sex for children. Moreover, if a woman has enough children, the intention for the next pregnancy will decrease and this leads to the likelihood of reporting unintended pregnancies. Surprisingly, the findings of Ameyaw [25] and Nyarko [26] on the relationship between parity and unintended pregnancy among women in Ghana contradicted the findings of the current study, although the current study shares some similarities with their studies in terms of the study population and variables. As explained earlier, the plausible reason for the disparity in finding could be explained by the role reproductive health decision-making plays in unintended pregnancy, a variable that was missing in the studies of Ameyaw [25] and Nyarko [26].

### Strengths and limitations

The use of a nationally representative survey (DHS) and the use of a stratified two-stage sampling technique made it possible to obtain a sample that is highly representative of the target population, that is, Ghanaian women in their reproductive age group. The use of relatively large sample size and the national representative nature of the data make conclusions from our study valid and generalisable. Nonetheless, the study sample was limited to only women in their reproductive age group (15–49 years old). Taking into consideration the socio-cultural connotations surrounding sex, there is also the possibility of social desirability bias due to the fact that women were asked about their sexual behavior. Again, the cross-sectional nature of the study does not make it possible to make any causal inference but rather only associations can be made. The answers to the main independent variable—reproductive health decision-making capacity—relied mainly on verbal report that was given by the women, without validating them from their partners.

## Conclusions and policy implications

Capitalizing on the gap that existed in literature with regard to the influence of reproductive health decision-making capacity on unintended pregnancies, this study has revealed the relationship that exists between reproductive health decision-making capacity and unintended pregnancies in Ghana. The findings from this study have highlighted the socio-demographic characteristics that influence unintended pregnancies in Ghana. This study has filled the gap in the already existing literature on unintended pregnancy in Ghana. Specific interventions geared towards reducing unintended pregnancies need to be directed toward women who do not have the capacity to make reproductive health decisions as well as women aged 15–19 years, those with primary education, Traditionalists, unmarried women, women from the Central, Greater Accra, Eastern, Volta, and Ashanti region and women with higher parity. For example, the Reproductive and Child Health Unit of the Ghana Health Service, in collaboration with non-governmental organisations dedicated to reproductive health issues, must intensify efforts to educate women and enhance their decision-making capacity on sexual intercourse and condom use by making them understand their sexual rights and giving them information and access to family planning services. Unlike this study, future research can focus on reproductive health decision-making capacity and unintended pregnancies in sub-Saharan Africa. Other studies could use the qualitative method to gain a deeper understanding of how reproductive health decision-making capacity can contribute to unintended pregnancy.
